# Thymoquinone Inhibits Autophagy and Induces Cathepsin-Mediated, Caspase-Independent Cell Death in Glioblastoma Cells

**DOI:** 10.1371/journal.pone.0072882

**Published:** 2013-09-09

**Authors:** Ira O. Racoma, Walter Hans Meisen, Qi-En Wang, Balveen Kaur, Altaf A. Wani

**Affiliations:** 1 Department of Radiology, The Ohio State University Wexner Medical Center, Columbus, Ohio, United States of America; 2 Department of Neurological Surgery, The Ohio State University Wexner Medical Center, Columbus, Ohio, United States of America; The University of Texas MD Anderson Cancer Center, United States of America

## Abstract

Glioblastoma is the most aggressive and common type of malignant brain tumor in humans, with a median survival of 15 months. There is a great need for more therapies for the treatment of glioblastoma. Naturally occurring phytochemicals have received much scientific attention because many exhibit potent tumor killing action. Thymoquinone (TQ) is the bioactive compound of the *Nigella sativa* seed oil. TQ has anti-oxidant, anti-inflammatory and anti-neoplastic actions with selective cytotoxicity for human cancer cells compared to normal cells. Here, we show that TQ selectively inhibits the clonogenicity of glioblastoma cells as compared to normal human astrocytes. Also, glioblastoma cell proliferation could be impaired by chloroquine, an autophagy inhibitor, suggesting that glioblastoma cells may be dependent on the autophagic pathway for survival. Exposure to TQ caused an increase in the recruitment and accumulation of the microtubule-associated protein light chain 3-II (LC3-II). TQ also caused an accumulation of the LC3-associated protein p62, confirming the inhibition of autophagy. Furthermore, the levels of Beclin-1 protein expression were unchanged, indicating that TQ interferes with a later stage of autophagy. Finally, treatment with TQ induces lysosome membrane permeabilization, as determined by a specific loss of red acridine orange staining. Lysosome membrane permeabilization resulted in a leakage of cathepsin B into the cytosol, which mediates caspase-independent cell death that can be prevented by pre-treatment with a cathepsin B inhibitor. TQ induced apoptosis, as determined by an increase in PI and Annexin V positive cells. However, apoptosis appears to be caspase-independent due to failure of the caspase inhibitor z-VAD-FMK to prevent cell death and absence of the typical apoptosis related signature DNA fragmentation. Inhibition of autophagy is an exciting and emerging strategy in cancer therapy. In this vein, our results describe a novel mechanism of action for TQ as an autophagy inhibitor selectively targeting glioblastoma cells.

## Introduction

Glioblastoma is a grade IV glioma and remains the most aggressive and devastating cancer of the central nervous system [1]. It is the most common brain tumor diagnosed in adults, with about 9,000 new diagnoses annually in the United States alone. Adding to this statistic is the number of recurring tumors, which occurs in a vast majority of cases. The standard of care for newly diagnosed glioblastoma is surgical resection of the tumor, followed by radiation therapy with concomitant and adjuvant chemotherapy with the alkylating agent temozolomide (TMZ). Despite this and other medical advances in the treatment of glioblastoma, the median survival time for patients is approximately 15 months from the first diagnosis. The molecular alterations that promote tumorigenesis and sustained growth of glioblastoma also serve to promote resistance to apoptosis [2], [3]. In recurrent glioblastomas, anti-apoptotic Bcl-2 and Bcl-X_L_ proteins of the Bcl-2 family are up-regulated, but the pro-apoptotic Bax and Bak proteins are down-regulated. This suggests that glioblastomas might naturally be under a selection pressure to develop resistance to apoptosis [2]. Another anti-apoptotic protein Bcl-2L12 is found to be up-regulated in almost all glioblastomas and contributes to apoptosis resistance by inhibiting caspase activation [2].

Recent studies concerning a number of different tumors, including glioblastoma [4]–[6] have alluded to the fact that cancer cells are significantly more dependent on autophagy for survival than non-cancer cells [7]–[11]. Autophagy is a lysosomal-dependent degradation system that functions to maintain cellular homeostasis by recycling unneeded proteins, eliminating defective organelles, and sustaining cell growth during brief periods of starvation and other stressors [12], [13]. It has been suggested that many oncoproteins such as the previously mentioned anti-apoptotic members of the Bcl2 family, phosphatidylinositol 3-kinase, and Akt suppress any autophagy beyond basal levels. However once a tumor has formed, autophagy is activated as a means to generate ATP and overcome the metabolic stress of the tumor environment [7], [14]. Additionally, many anti-cancer drugs up-regulate autophagy, which can lead to recalcitrant tumors [9], [15], [16]. Recent studies have demonstrated that pharmacological or genetic inhibition of autophagy enhances the effects of conventional radio- and chemotherapy [7], [11], [17], suggesting that inhibition of autophagy might be a viable and auspicious strategy for cancer treatment. At the moment, chloroquine (CQ) and its derivative hydroxychloroquine (HCQ), which have both been used for years as anti-malarial and anti-rheumatoid arthritis drugs, are the only autophagy inhibitors in clinical trials for cancer therapy [4], [18]. CQ and HCQ are lysosomotropic agents, thereby preventing lysosome acidification and subsequent fusion of the autophagosome with the lysosome. However, long-term administration of chloroquine can result in retinopathies [19] which may limit its use as a chemotherapeutic.

There has been a growing interest in natural compounds with anti-cancer properties precisely because they are relatively non-toxic to healthy cells and are available in a readily-ingested form. The dietary phytochemical thymoquinone (TQ) is the primary bioactive component of *Nigella sativa* Linn seed (also known as black seed) oil. *Nigella sativa* has been used for centuries in Middle Eastern, Indian and European countries for culinary purposes and to promote good health [20], with the beneficial properties being attributed to TQ [21]. Its purported health benefits include anti-inflammatory, anti-oxidant and anti-hypertensive actions. We have shown that TQ induces apoptosis in HL-60 leukemia cells and MCF-7/DOX doxorubicin-resistant breast cancer cells [22], [23]. Additionally, a number of other studies have reported that the anti-tumor functions of TQ are specific for cancer cells [24], [25]. Pharmacokinetic studies have shown that mice and rats can consume large amounts of TQ without adverse effects [26]. Importantly, TQ readily crosses the blood-brain barrier due to its small size and lipophilicity [26], [27].

Here we report a novel mechanism of action for TQ which involves the inhibition of autophagy in glioblastoma cells via perturbation of the lysosomal membrane and cathepsin translocation from the lysosomal lumen to the cytosol, leading to caspase-independent apoptosis.

## Materials and Methods

### Cell culture and reagents

Human glioblastoma cells T98G and U87MG were kindly provided by Dr. Arnab Chakravarti, purchased from ATCC (ATCC numbers CRL-1690 and HTB-14, respectively). T98G and U87MG cells were grown in DMEM (Life Technologies, Carlsbad, CA) supplemented with 10% fetal bovine serum (Atlanta Biologicals, Lawrenceville, GA) and 0.05% penicillin/streptomycin (Life Technologies). Human glioblastoma cells Gli36ΔEGFR containing a mutant EGFR (vIII) [28], [29] were kindly provided by Dr. Balveen Kaur, and were grown in DMEM supplemented with 10% fetal bovine serum, 0.05% penicillin/streptomycin, 2 µg/ml puromycin (Sigma, St. Louis, MO) and 4 µg/ml blasticidin (Sigma). Normal human astrocytes (NHA) were grown in Complete Astrocyte Medium (kit), both purchased from ScienCell Research Laboratories (Carlsbad, CA) on poly-l-lysine coated plates (ScienCell Research Laboratories). All cells were grown at 37°C in a humidified 5% CO_2_ atmosphere. Thymoquinone, chloroquine, acridine orange and MTT were purchased from Sigma. Cathepsin inhibitor III (peptide sequence: Z-Phe-Gly-NHO-Bz-*p*OMe) was purchased from EMD Chemicals (Darmstadt, Germany). z-VAD-FMK was purchased from R&D Systems (Minneapolis, MN).

### Clonogenic cell-survival assays

Cells were plated at a low density and allowed to attach for 4–6 hours before treating with increasing concentrations of TQ or chloroquine. Cells were allowed to grow until colonies contained >50 cells per colony (7–10 days). After the growth period, cells were fixed with 3.7% paraformaldehyde for 15 minutes at room temperature, followed by staining with 1% methylene blue, also for 15 minutes at room temperature. Colonies were defined as having >50 cells and were manually counted. The surviving fraction (SF) for each treatment was calculated as a ratio of the number of colonies counted to the number of cells plated multiplied by plating efficiency. Lines were generated through best fit non-linear regression automatic analysis with TableCurve software (Systat Software Inc., San Jose, CA). Alternatively, cells were also plated at a low density, allowed to attach for 4–6 hours prior to treating with TQ. Cells were grown until untreated controls were confluent, then they were fixed with 3.7% paraformaldehyde and stained with 1% methylene blue as described above, air dried, photographed and evaluated for colony estimation.

### Cell viability assay

Quantitation of cell viability was determined by the MTT assay. Cells were seeded in 96-well plates, pre-treated with Z-VAD-FMK (10 or 20 µM) or cathepsin inhibitor III cocktail (5 or 10 µM) for 1 hour followed by TQ (20 µM or 40 µM) for 24 hours. MTT solution was then added to the cultures to a final concentration of 0.5 mg/ml and incubated at 37°C for an additional 4 hours. The medium was aspirated and the formazan crystals were solubilized with DMSO after which the absorbance was determined by a uQuant microplate reader (Biotek Instruments, Winooski, VT) at 570 nm.

### Apoptosis assay

Apoptosis was determined by flow cytometric analysis of PI and Annexin V stained cells. Cells were treated with increasing concentrations of TQ for 6 hours, washed and pelleted. Each sample was resuspended in 100 µl of staining solution composed of Annexin V (Molecular Probes, Carlsbad, CA) and propidium iodide (Sigma) in assay binding buffer, according to manufacturer's instructions. Cells were incubated in the dark at room temperature for 15 minutes and immediately analyzed by an Aria III flow cytometer (BD Biosciences, San Jose, CA).

### DNA fragmentation analysis

Cells were treated with increasing concentrations of TQ for 24 hours. Detailed procedure for DNA fragmentation analysis has been described previously [23]. Briefly, adherent and floating cells were pooled together and lysed. RNA and protein were degraded by incubation with RNase and Proteinase K, respectively. DNA was precipitated with 100% ethanol and resuspended in TE buffer, pH 8. DNA was evaluated for fragmentation by resolving on a 1% agarose gel, stained with ethidium bromide and visualized under UV light.

### Western blot analysis

Cells were treated with TQ, chloroquine or a combination of the two for 24 hours. Cells were harvested and lysed as previously described [23]. 50 µg of total protein for each sample was separated by SDS-PAGE as follows: LC3 was resolved with a 12% gel; p62/SQSTM1 and Beclin-1 were resolved with an 8% gel. Proteins were transferred to PVDF membranes and blocked with 5% non-fat dry milk in TBST buffer (blocking buffer) at 37°C for one hour. Membranes were incubated in primary antibody at 4°C overnight with rocking, followed by washing three times with TBST buffer then incubated with the appropriate HRP-conjugated secondary antibody at 37°C for one hour. The membranes were washed three times with TBST buffer and examined by chemiluminescence detection.

Antibodies against the following proteins were diluted in blocking buffer: β-actin, diluted 1/5000 (Santa Cruz Biotechnology, Santa Cruz, CA); microtubule associated light chain 3 (LC3), diluted 1/1000; p62/SQSTM1, diluted 1/1000; and Beclin-1, diluted 1/1000 (all from Cell Signaling Technology, Danvers, MA).

### Acridine orange staining

Acidic lysosomes were labeled with acridine orange. Cells were grown on coverslips and treated with TQ or chloroquine for 1 or 6 hours. After treatment, cells were washed once with PBS containing Ca^2+^ and Mg^2+^ and stained with 1 µM acridine orange at 37°C for 15 minutes [30], [31]. Excess acridine orange was washed away with PBS and cells were immediately examined on a Nikon Eclipse 80i (Nikon Instruments Inc., Melville NY) wide field fluorescent microscope.

### Statistical Analysis

Statistical significance of differences observed between TQ-treated and TQ in combination with either Z-VAD-FMK or cathepsin inhibitor were determined using the Student's t-test. Differences were considered to be significant at p<0.05.

## Results

### TQ inhibits glioblastoma cell proliferation

**Figure 1:**
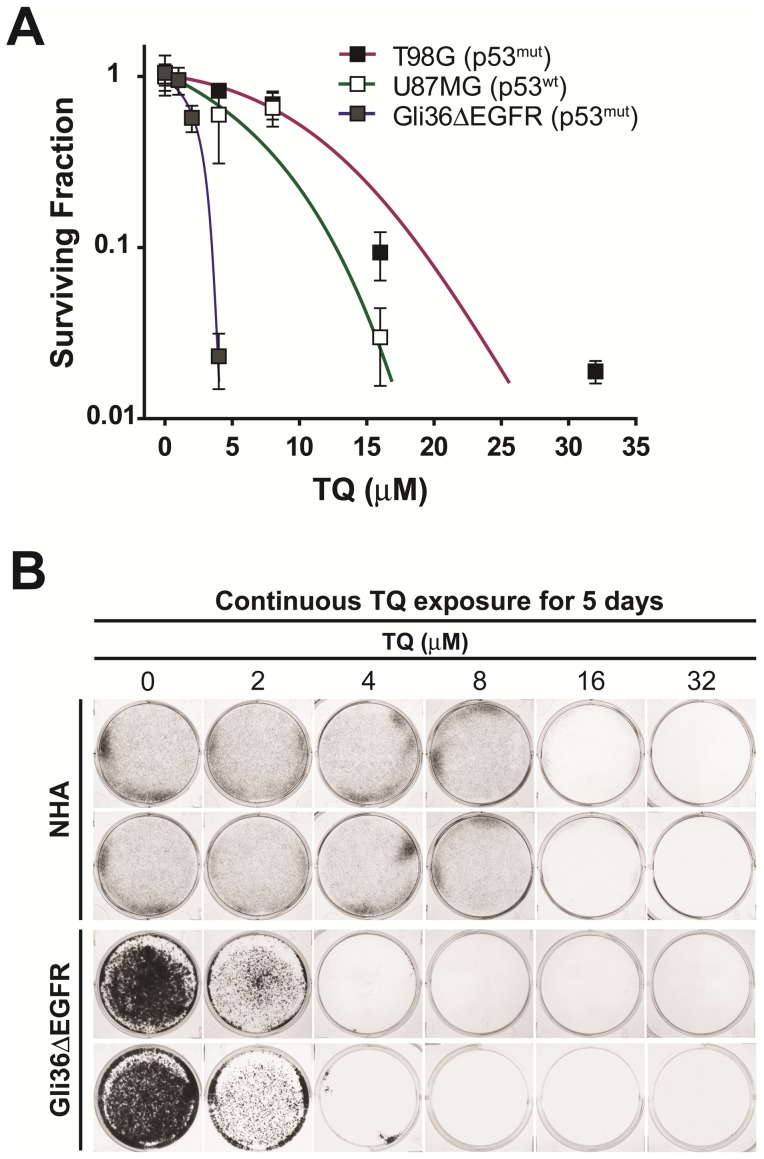
TQ inhibits glioblastoma cell proliferation. A) T98G, U87MG and Gli36ΔEGFR cells were incubated with increasing concentrations of TQ for 7–10 days. At the end of the growth period colonies were fixed, stained and counted. After taking into account the plating efficiency for each cell line, the surviving fraction is the ratio of the number of colonies counted and the initial number of cells plated. Mean ± S.D., n = 3. B) The effect of TQ on proliferation of normal human astrocytes (NHAs) compared to Gli36ΔEGFR cells was determined by a modified clonogenic assay. Cells were plated at a low density and incubated with increasing concentrations of TQ for 5 days. At the end of the growth period, cells were fixed, stained and photographed for estimation of proliferation relative to untreated cells.

TQ has shown to be effective in killing a number of different tumor types. To test the effect of TQ on glioblastoma cell proliferation, we subjected a heterogeneous set of glioblastoma cells to a clonogenicity based cell survival assay, which accurately assesses the inhibition of cell proliferation in response to a cytotoxic or cytostatic agent. T98G (p53^mut^), U87MG (p53^wt^) and Gli36ΔEGFR (p53^mut^) cells were plated at a low density and incubated with TQ for 7–10 days. After the growth period, the colonies formed were fixed and counted, and represented as a surviving fraction of the initial number of cells seeded. TQ prevented glioblastoma cell proliferative capacity in the three cell lines we tested, albeit to varying degrees (Figure 1A). For example, Gli36ΔEGFR cells were found to be the most sensitive to TQ (IC_50_ = 2.4 µM), while T98G cells were the most resistant (IC_50_ = 10.3 µM) and U87MG cells exhibited intermediate sensitivity (IC_50_ = 8.3 µM). The sensitivity of each cell line does not appear to correlate with p53 status, which is in agreement with earlier studies showing that TQ is cytotoxic to cancer cells independent of p53 status [22], [32]. We also found that normal human astrocytes were not sensitive to TQ by comparing their growth against Gli36ΔEGFR cells. The survivability of diffusely growing NHA could not be determined from colony formation as they fail to produce distinct countable colonies. Their growth inhibition was determined from visual inspection of cell populations against varying TQ concentrations. As can be seen in Figure 1B, NHA proliferation was not affected at least up to 8 µM TQ with some easily visible cell growth even at TQ concentrations up to 16 µM. On the other hand, 2 µM of TQ was enough to drastically inhibit growth of Gli36ΔEGFR cells, with no visible cells at 4 µM. These results are in full agreement with the Gli36ΔEGFR colony formation results in Figure 1A.

### Autophagy inhibition prevents glioblastoma cell proliferation

**Figure 2:**
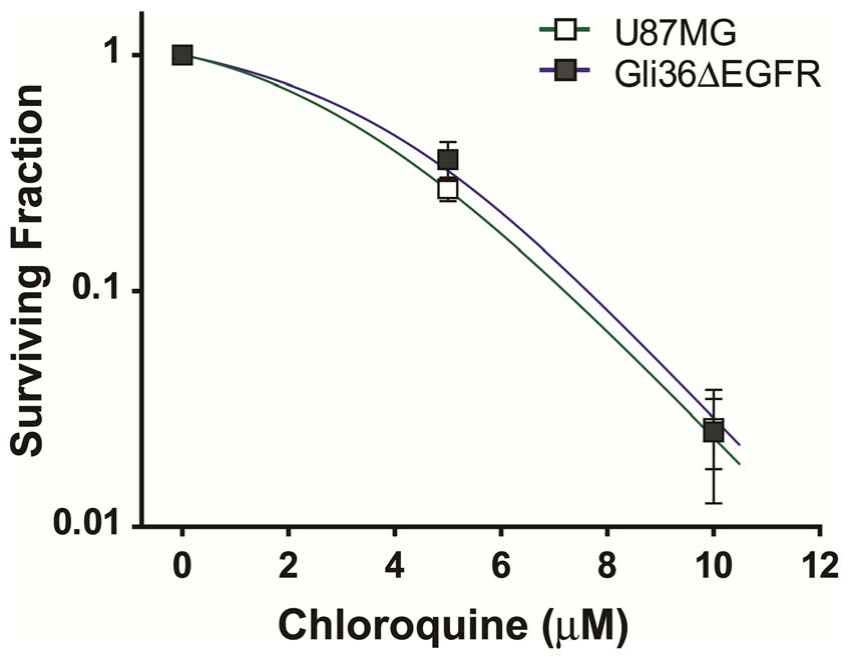
Chloroquine inhibits glioblastoma cell proliferation. U87MG and Gli36ΔEGFR cells were incubated with increasing concentrations of CQ for 7–10 days. At the end of the growth period colonies were fixed, stained and counted. After taking into account the plating efficiency for each cell line, the surviving fraction is the ratio of the number of colonies counted and the initial number of cells plated. Mean ± S.D., n = 3.

Once a tumor has formed, they have been shown to be more dependent on autophagy as a means to survive the metabolic stress of the tumor environment [33], [34]. Pharmacological or genetic inhibition of autophagy is reported to induce cell death in cancer cells. Therefore, we wanted to determine the sensitivity of glioblastoma cell proliferation to pharmacologic inhibition of autophagy with chloroquine. We performed a clonogenic assay as described above for TQ with increasing concentrations of chloroquine and found that chloroquine reduced the proliferative capacity of U87MG and Gli36ΔEGFR cells to a similar extent (IC_50_ = 3.6 µM and 4.4 µM, respectively) in each cell line (Figure 2).

### Thymoquinone inhibits autophagic flux in glioblastoma cells

**Figure 3:**
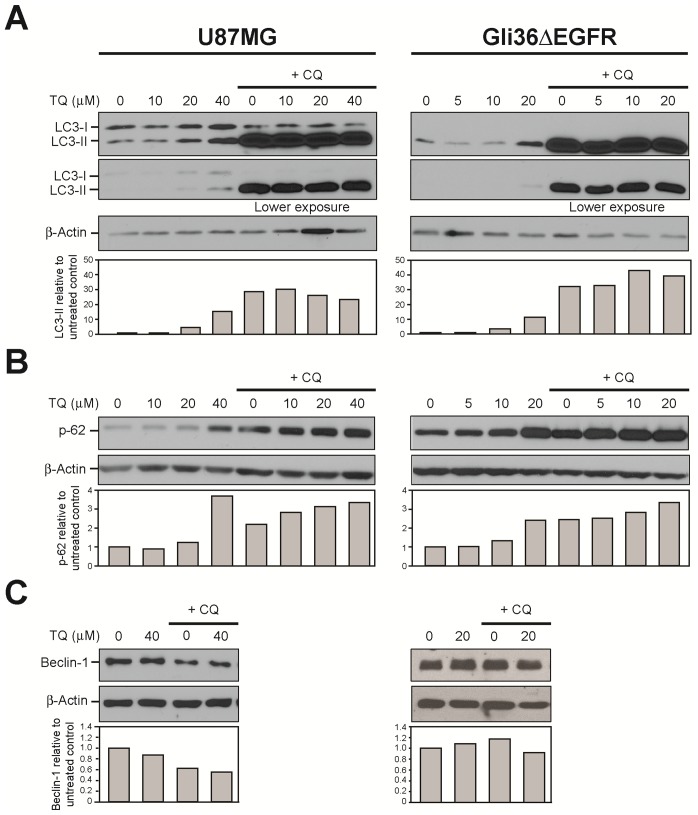
TQ blocks autophagy in glioblastoma cells. U87MG and Gli36ΔEGFR cells were treated with increasing concentrations of TQ, with and without chloroquine (40 µM), harvested at 24 hours and immunoblotted for A) microtubule-associated light chain (LC3-I and LC3-II), and B) p62. C) Cells were treated with TQ, with and without chloroquine, harvested at 1 hour and immunoblotted for Beclin-1. LC3-II, p62 and Beclin-1 levels were normalized to actin. Graphs show protein levels relative to untreated control.

Autophagic flux is defined as the complete process of autophagy beginning with formation of the autophagosome around the cargo, followed by fusion of the autophagosome with the lysosome, then ending with degradation and recycling of the cargo [12], [35]. To investigate the effect of TQ on autophagic flux, we treated U87MG and Gli36ΔEGFR cells with TQ, chloroquine or a combination of the two and subjected whole cell lysates to Western blotting to determine changes in expression of LC3-II, p62 and Beclin-1. Microtubule-associated protein 1 light chain 3, or LC3, a ubiquitin-like protein, is synthesized in an unprocessed form called pro-LC3. Proteolytic processing yields the 16 kDa form called LC3-I, which can then be conjugated to phosphatidyl ethanolamine (PE) during autophagy to yield the 14 kDa form called LC3-II. LC3-II is widely used as a marker for complete autophagosomes [35]–[37]. As shown in Figure 3A, treatment with increasing concentrations of TQ for 24 hours resulted in a dose-dependent increase of LC3-II. Treatment with chloroquine alone greatly increased LC3-II levels. A combination of chloroquine and increasing concentrations of TQ resulted in more LC3-II compared to TQ alone, which may be due to either autophagy induction or autophagy inhibition. However, combination treatment compared to chloroquine alone did not result in an increase of LC3-II, suggesting that TQ is blocking autophagy.

To further explore the effect of TQ on autophagic flux, we also measured the changes in p62 levels. p62, which is also known as SQSTM1, is a ubiquitin-binding protein that is involved in lysosome- or proteasome-dependent degradation of proteins. It incorporates into the autophagosome via direct interaction with LC3-II and is degraded in the process of autophagy. Inhibition of autophagy leads to increased levels of p62 [35], [36]. As shown in Figure 3B, treatment with increasing concentrations of TQ for 24 hours resulted in a dose-dependent increase of p62 levels. Furthermore, combination treatment with chloroquine and increasing concentrations of TQ resulted in a dose-dependent increase of p62 levels compared to either TQ or chloroquine alone, confirming that TQ inhibits autophagic flux in these glioblastoma cells. In further support of TQ as an autophagy inhibitor, we measured the levels of Beclin-1. Beclin-1 levels are associated with autophagy induction, as it is needed to initiate autophagosome formation [12]. After 1 hour treatment with TQ, Beclin-1 levels do not change, providing additional evidence of TQ as an autophagy inhibitor (Figure 3C).

### Lysosomal membrane permeabilization (LMP) and cathepsin activity is involved in thymoquinone-induced cell death

**Figure 4:**
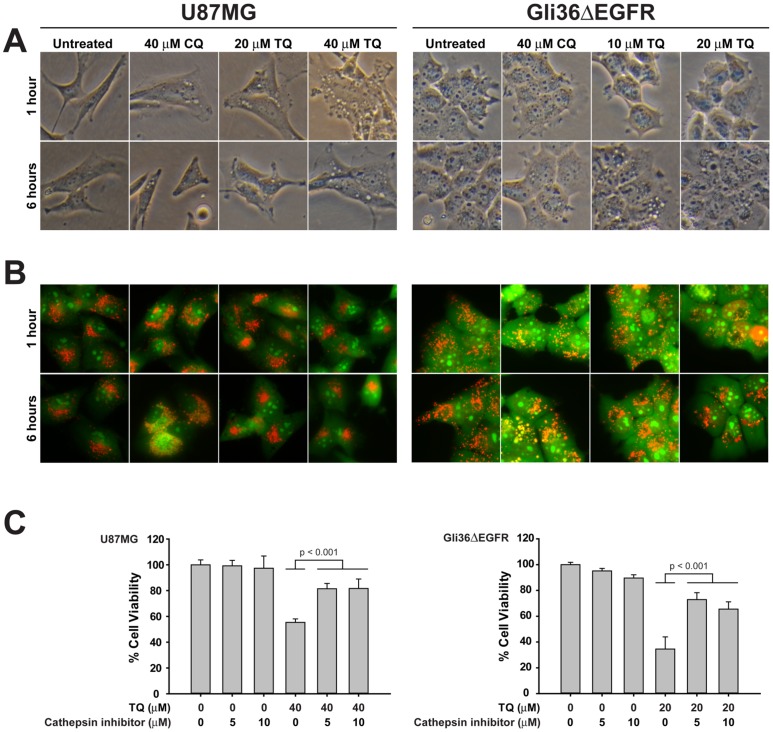
Lysosomal membrane permeabilization (LMP) and cathepsin activity is involved in thymoquinone-induced cell death. A) Cytoplasmic vacuolization was observed in U87MG cells after treatment for 1 hour with either TQ or chloroquine, but not in Gli36ΔEGFR cells. At 6 hours, vacuolization can be seen in both cell types. B) Treatment with TQ or chloroquine for 6 hours induces lysosome membrane permeability in U87MG and Gli36ΔEGFR cells, seen as the loss of red acridine orange staining. C) Cells were pre-treated with cathepsin inhibitor III, which primarily targets cathepsin B, for 1 hour. TQ was added and incubated with the cells for 24 hours. Cell viability was measured using the MTT assay.

Increased expression of LC3-II and p62, taken together with stable levels of Beclin-1, strongly indicate that TQ inhibits a later stage of autophagy, particularly at the point of autophagosome-lysosome fusion. Upon inhibition of autophagy at this stage, cells begin to display cytoplasmic vacuolization [38], which has been reported by a number of studies [38]–[41]. Consistent with these findings, chloroquine and TQ promote cytoplasmic vacuolization in both U87MG and Gli36ΔEGFR cells at 6 hours, but can be seen as early as 1 hour in U87MG cells (Figure 4A). 40 µM CQ induced less vacuolization in both cells lines compared to TQ. In U87MG cells, both concentrations of TQ induced the same extent of vacuolization. In Gli36ΔEGFR cells, 20 µM TQ caused more, but smaller vacuoles than 10 µM TQ. An apparently anomalous dose-dependent change in vacuole size has been observed previously in macrophages in which 100 µM chloroquine causes smaller vacuolization than 30 µM [42].

Defective autophagosome-lysosome fusion can be attributed to a perturbation of lysosomal activity [43]. Therefore, we sought to evaluate the effect of TQ on the lysosome by first examining lysosome membrane permeability (LMP). Disruption of the lysosomal membrane can play a major role in programmed cell death by mediating both caspase dependent and independent cell death. To assess the effect of TQ on lysosomal membrane permeability (LMP), lysosomes were labeled with acridine orange (AO). AO is a weakly basic lysosomotropic dye that has differential staining capabilities. At a low pH, AO will fluoresce orange-red, but at neutral pH it will fluoresce green. Untreated cells prominently display punctate orange-red staining of lysosomes throughout the cytoplasm, while in CQ-treated cells there is a marked decrease in orange-red staining. After TQ treatment, while there is a modest decrease in red acridine orange staining in U87MG cells,Gli36ΔEGFR cells exhibited a considerable decrease of red staining(Figure 4B). Furthermore, TQ appears to be more effective at destabilizing the lysosomal membrane than the well-characterized lysosomotropic agent chloroquine. TQ treatment also appears to affect lysosome localization. In untreated cells, lysosomes can be seen dispersed throughout the cytosol and at the edges of the cells, whereas treatment with TQ appears to cause the remaining intact lysosomes to cluster around the nucleus. Cancer cells often display peripheral localization of lysosomes, particularly those at the invasive edges of tumors [38] however, non-cancer cells typically have a perinuclear arrangement of lysosomes.

A direct consequence of LMP is the translocation of cathepsin proteases and other hydrolytic enzymes from the lysosomal lumen to the cytosol [38], [44]. While all have optimal activity at the low pH inside lysosomes, a few, including the cysteine cathepsins, are still functional at neutral pH [45] and capable of signaling to downstream mediators of cell death. To evaluate the contribution of cathepsin activity to TQ-mediated cell death, U87 and Gli36ΔEGFR cells were incubated with TQ, either with or without a cathepsin inhibitor cocktail (Cathepsin inhibitor III, EMD Millipore), which primarily targets the cysteine cathepsin B, for 24 hours. As shown in Figure 4C, the presence of a cathepsin inhibitor significantly increased the percentage of viable TQ-treated cells compared to TQ treatment alone.

### Thymoquinone induces caspase-independent apoptotic cell death in glioblastoma cells

**Figure 5:**
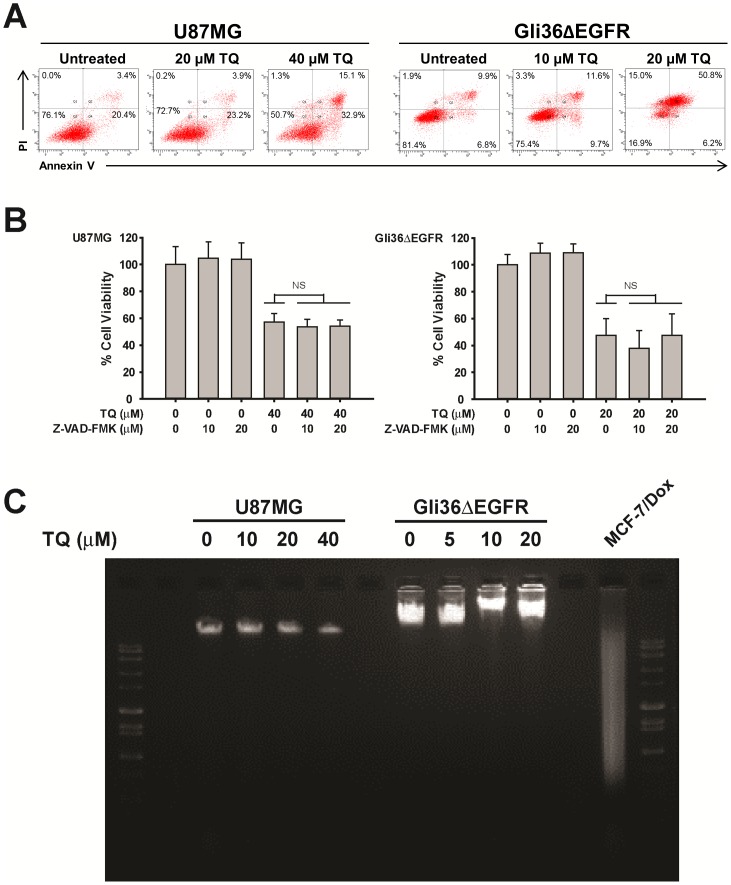
TQ induces caspase-independent apoptosis. A) Cells were treated with increasing concentrations of TQ for 6 hours and analyzed by flow cytometry for PS flipping, as determined by an increase in Annexin V positive cells. Cells were co-labeled with propidium iodide to evaluate cell viability. B) Cells were pretreated with the general caspase inhibitor z-VAD-FMK for 1 hour. TQ was added and incubated with the cells for 24 hours. Cell viability was measured using the MTT assay. Mean ± S.D., n = 3 C) Cells were treated with increasing concentrations of TQ for 24 hours then evaluated for DNA fragmentation through agarose gel electrophoresis.

One of the hallmarks of glioblastoma cells is their resistance to apoptotic cell death following radiation and chemotherapy [46]–[50]. This is thought to be a direct result of upregulated survival signaling and increased expression of anti-apoptosis proteins. As TQ has been shown to induce apoptosis in a variety of cancer cells, we sought to determine whether TQ can also induce apoptosis in glioblastoma cells by labeling cells with propidium iodide and Annexin V to detect phosphatidyl serine (PS) flipping. PS is localized to the cytoplasmic face of the cell membrane in healthy cells, but translocates to the extracellular face of the cell membrane in apoptotic cells and thus can be detected by conjugation to Annexin V. This translocation process occurs relatively early in the cell death program. After treating cells with increasing concentrations of TQ, a dose dependent increase in PI and Annexin V double positive cells was observed. In U87MG cells, approximately 15% of the cells are double positive for PI and Annexin V after treatment with TQ for 6 hours (Figure 5A). The effect is even more dramatic in Gli36ΔEGFR cells, as approximately 50% of the cells are undergoing apoptosis (Figure 5A). Interestingly, the apoptotic program in glioblastoma cells appears to be caspase independent. We pre-treated cells for 1 hour with the general caspase inhibitor z-VAD-FMK prior to incubation with TQ for an additional 24 hours. Cell viability was then measured with the MTT assay. As expected, TQ alone reduced the cell viability of U87MG cells by approximately 43%, and of Gli36ΔEGFR cells by approximately 53%. Once again, however, pre-treatment with either 10 µM or 20 µM z-VAD-FMK failed to revert the viability of either cell line in the presence of TQ (Figure 5B), although this is within the effective concentration range of this general caspase inhibitor [51]. We evaluated the degree of DNA fragmentation in glioblastoma cells after treatment with increasing concentrations of TQ. The degradation of DNA into fragments of multiples of 180 base pairs is carried out by certain caspase-activated DNases, and is an essential feature of classical apoptotic cell death. After treating with increasing concentrations of TQ, mostly high molecular weight virtually intact DNA was recovered from U87MG and Gli36ΔEGFR cells, similar to what is seen in the untreated controls (Figure 5C). The results for both cell lines are in contrast to the extensive fragmentation observed in TQ-treated doxorubicin-resistant MCF-7 cells, which we have previously reported to die specifically by apoptosis in a caspase-dependent manner upon TQ treatment [23].

## Discussion

Glioblastoma is the most common and the most aggressive malignant astrocytic brain tumor in adults. The median survival time from the first diagnosis is approximately 15 months. Tumor resection followed by ionizing radiation (IR) therapy and chemotherapy with the alkylating agent temozolomide (TMZ) is the standard of care for glioblastoma, however a majority of patients will experience a recurrence of the tumor around the same location as the original tumor. Tumors recur for several reasons: glioblastomas are highly infiltrative and diffuse, therefore de-bulking cannot remove all of the initial cancer cells; they have a high degree of molecular heterogeneity, therefore radiation and chemotherapy will not kill the entire population of diverse cells of a single tumor. In fact, when combined with IR, TMZ is only successful in the subset of patients who carry a methylated *O^6^*-methylguanine-DNA methyltransferase (MGMT) promoter [52].

**Figure 6:**
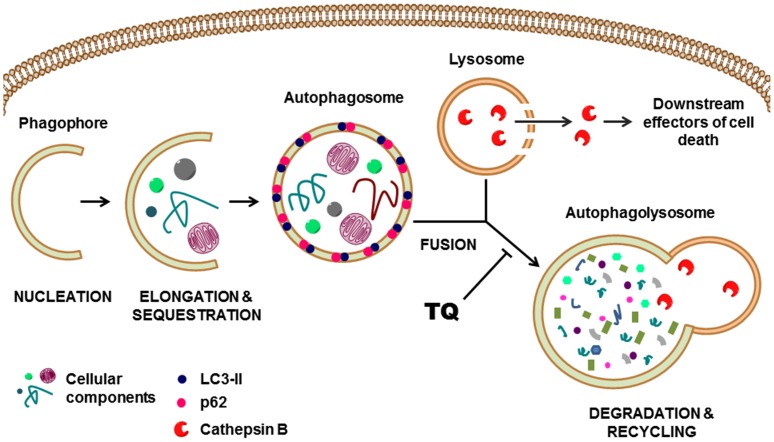
Inhibition of late stage autophagy by TQ. Pathway shows initiation of autophagosome formation, however, LC3-II and p62 accumulate due to TQ-mediated decrease in autophagosome degradation. TQ also mediates lysosome permeability resulting in leakage of cathepsin B into the cytosol where it can signal to downstream mediators of cell death.

Thymoquinone (TQ) is the most abundant bioactive component of the *Nigella sativa* (black cumin) seed oil. We and others [22], [23], [32], [53] have previously shown that TQ induces apoptosis in several experimental cancer models. In the present study, we describe a novel mechanism of action for TQ in apoptosis-resistant glioblastoma cells and show that TQ inhibits the autophagic flux by inducing lysosome membrane permeabilization and subsequent translocation of lysosomal hydrolases to the cytosol (Figure 6).

A clonogenic assay revealed that TQ inhibits growth in three different glioblastoma cells to different extents; however the p53 status of the cells does not appear to affect the mechanism by which TQ inhibits growth and subsequent colony formation. This is not surprising as TQ has been reported to be cytotoxic to p53-wild type [32] and p53-mutant [22] tumor cells. We also found that growth of normal human astrocytes is not affected by concentrations of TQ that are cytotoxic to glioblastoma cells. This is in agreement with previous reports showing that TQ is selective for cancer cells [24], [25], [54]. It is important to note that TQ readily crosses the blood brain barrier: in mouse models of petit mal epilepsy, TQ administered by intraperitoneal injection was shown to delay the onset and decrease the duration of seizures [26], and in rat models of ischemia-induced brain injury, TQ administered in drinking water prevented the depletion of several crucial endogenous neuronal antioxidants [27]. Given these precedents in *in vivo* models of two different neurological pathologies, and considering the chemical properties of TQ, it is reasonable to expect that TQ may provide a therapeutic benefit in both animal models of glioblastoma and in patients.

Here, we show that glioblastoma cells are sensitive to growth inhibition by chloroquine. Several studies have reported on the enhancing effect of chloroquine on traditional chemotherapies and on ionizing radiation therapy [7], [11], [17], including a small, randomized, double blind and placebo controlled trial in which the median survival for glioblastoma patients receiving chloroquine in addition to conventional radiation and chemotherapy was 24 months, compared to the placebo group for whom median survival was 11 months [4]. Chloroquine and its analog hydroxychloroquine accumulate in the lysosome as they become protonated within the acidic environment and cannot diffuse back out. The ability for chloroquine alone to cause a dose-dependent inhibition in glioblastoma cells strongly suggests that these cells are highly dependent on autophagy. Single-treatment with chloroquine was found to induce both apoptosis and necrosis, depending on the concentration, in A549 lung cancer cells [55].

TQ causes a time- and dose-dependent formation of cytoplasmic vacuoles. Many drugs, including chloroquine, neutral red, propranolol, atropine, and lidocaine, induce cytoplasmic vacuolization [42], [43]. Upon exposure to cytotoxic compounds, cells will attempt to sequester the drug into vacuoles to protect themselves. Vacuoles disappear after removal of the drug from the cell culture media, but prolonged vacuolization of the lysosome might lead to irreversible changes that result in cell death, particularly the release of a cationic form of the drug back into the cytosol. This implies that the lysosome membrane is now permeable [43].

We then demonstrate that TQ causes lysosome membrane permeability (LMP) via a decrease in red acridine orange staining. An obvious corollary to LMP is the leakage of lysosomal proteases, such as cathepsins, from the lysosome to the cytosol. While most cathepsins are non-functional at the relatively neutral pH of the cytosol, several, namely cathepsins B and D, are still active and capable of signaling to downstream mediators of cell death such as the mitochondria [44]. In some cell types, released cathepsins can induce apoptotic cell death [56], but in apoptosis-resistant cells they mediate caspase-independent cell death [56], [57]. Furthermore, cathepsin B is overexpressed in gliomas and serves as a strong prognostic marker for primary tumors of the CNS [58]. While this change allows for a potentiation of angiogenic and metastatic capacity of tumors, it also confers a susceptibility to lysosome membrane permeabilization [56] by chemotherapeutic agents. It is known that microtubule poisons, such as paclitaxel and vincristine, are capable of inducing LMP [38], [44], [57] and interfering with lysosomal trafficking [59]. TQ was recently demonstrated to target microtubules, causing a time and dose-dependent degradation of α and β-tubulin in U87MG glioblastoma and Jurkat T lymphoblastic leukemia cells [54]. Taken together, these reports support our findings that TQ induces LMP and cathepsin translocation in glioblastoma cells.

In agreement with other reports that TQ induces apoptosis in cancer cells, we also observed apoptosis induction by detection of PS flipping. Interestingly, the general caspase inhibitor z-VAD-FMK was unable to preserve cell viability, strongly pointing to a caspase-independent process. This was confirmed by the lack of DNA fragmentation after TQ treatment, as DNases are activated by caspases. Mechanistic studies of novel glioblastoma therapies involving the natural compound asiatic acid [47] and a retroviral approach using mutant survivin [48] have also reported that z-VAD-FMK could not protect glioblastoma cell death from these various insults. In addition to caspase-independent apoptosis, increasing evidence is pointing to the existence of other mechanisms of cell death, for example apoptosis-like cell death, in which caspase activation and other markers of classical apoptosis are completely absent [44], [57], and can help explain why inhibition of caspases does not always protect cells from cytotoxic stimuli. A number of non-caspase proteases such as lysosomal cathepsins are capable of executing programmed cell death. Additionally, release of mitochondrial proteins other than cytochrome c such as endonuclease G and apoptosis inducing factor (AIF) also play a role in caspase-independent programmed cell death [60].

Other labs have reported that the cytotoxicity of TQ is due to the *in vitro* generation of reactive oxygen species by virtue of its quinone chemical structure [61]. However, in these studies, N-acetyl-cysteine (NAC) was used to inhibit the pro-oxidant effects of TQ. This might have confounded their results because TQ is also an arylating quinone [62], and NAC might have formed an adduct with TQ before TQ had the opportunity to redox cycle. Further complicating the issue is that arylating quinones are capable of inducing cell death independent of ROS by initiating the unfolded protein response, and this phenomenon can be inhibited by pre-treating cells with NAC [63]. Nevertheless, ROS are known to induce LMP on their own [38], [44], and arylating/alkylating agents can disrupt microtubules [56] which results in LMP. Presently, we do not know which of these mechanisms is prevalent in TQ-induced LMP, and is deserving of further exploration.

Ionizing radiation and temozolomide have both been shown to increase a cytoprotective autophagy response in glioblastoma cells, leading to resistant tumors. In addition, many other chemotherapeutics, such as rapamycin, tamoxifen, and etoposide, induce a protective autophagic response in cancer cells. Therefore, inhibitors of autophagy, both alone and in combination with standard therapies, may provide a viable and promising new strategy in cancer treatment. The immunosuppressant FTY720 and the anti-schistome lucanthone (Miracil D), have both been identified as autophagy inhibitors in mantle cell lymphoma [64] and breast cancer [11], respectively. To the best of our knowledge, this report represents the first finding of TQ as an autophagy inhibitor, and provides a platform for which to extend studies in the treatment of glioblastoma with TQ.

## References

[1] Perry J, Okamoto M, Guiou M, Shirai K, Errett A, et al (2012) Novel therapies in glioblastoma. Neurol Res Int 2012: 428565. 22530121 10.1155/2012/428565 PMC3316989

[2] Krakstad C, Chekenya M (2010) Survival signalling and apoptosis resistance in glioblastomas: opportunities for targeted therapeutics. Mol Cancer 9: 135. 20515495 10.1186/1476-4598-9-135 PMC2893101

[3] Ziegler DS, Kung AL, Kieran MW (2008) Anti-apoptosis mechanisms in malignant gliomas. J Clin Oncol 26: 493–500. 18202424 10.1200/JCO.2007.13.9717

[4] Sotelo J, Briceno E, Lopez-Gonzalez MA (2006) Adding chloroquine to conventional treatment for glioblastoma multiforme: a randomized, double-blind, placebo-controlled trial. Ann Intern Med 144: 337–343. 16520474 10.7326/0003-4819-144-5-200603070-00008

[5] Katayama M, Kawaguchi T, Berger MS, Pieper RO (2007) DNA damaging agent-induced autophagy produces a cytoprotective adenosine triphosphate surge in malignant glioma cells. Cell Death Differ 14: 548–558. 16946731 10.1038/sj.cdd.4402030

[6] Lomonaco SL, Finniss S, Xiang C, Decarvalho A, Umansky F, et al (2009) The induction of autophagy by gamma-radiation contributes to the radioresistance of glioma stem cells. Int J Cancer 125: 717–722. 19431142 10.1002/ijc.24402

[7] Apel A, Herr I, Schwarz H, Rodemann HP, Mayer A (2008) Blocked autophagy sensitizes resistant carcinoma cells to radiation therapy. Cancer Res 68: 1485–1494. 18316613 10.1158/0008-5472.CAN-07-0562

[8] Bristol ML, Di X, Beckman MJ, Wilson EN, Henderson SC, et al (2012) Dual functions of autophagy in the response of breast tumor cells to radiation: cytoprotective autophagy with radiation alone and cytotoxic autophagy in radiosensitization by vitamin D 3. Autophagy 8: 739–753. 22498493 10.4161/auto.19313 PMC3378418

[9] Livesey KM, Tang D, Zeh HJ, Lotze MT (2009) Autophagy inhibition in combination cancer treatment. Curr Opin Investig Drugs 10: 1269–1279. 19943199

[10] Solomon VR, Lee H (2009) Chloroquine and its analogs: a new promise of an old drug for effective and safe cancer therapies. Eur J Pharmacol 625: 220–233. 19836374 10.1016/j.ejphar.2009.06.063

[11] Carew JS, Espitia CM, Esquivel JA 2nd, Mahalingam D, Kelly KR, et al (2011) Lucanthone is a novel inhibitor of autophagy that induces cathepsin D-mediated apoptosis. J Biol Chem 286: 6602–6613. 21148553 10.1074/jbc.M110.151324 PMC3057822

[12] Mizushima N (2007) Autophagy: process and function. Genes Dev 21: 2861–2873. 18006683 10.1101/gad.1599207

[13] Klionsky DJ, Abdalla FC, Abeliovich H, Abraham RT, Acevedo-Arozena A, et al (2012) Guidelines for the use and interpretation of assays for monitoring autophagy. Autophagy 8: 445–544. 22966490 10.4161/auto.19496 PMC3404883

[14] Morselli E, Galluzzi L, Kepp O, Vicencio JM, Criollo A, et al (2009) Anti- and pro-tumor functions of autophagy. Biochim Biophys Acta 1793: 1524–1532. 19371598 10.1016/j.bbamcr.2009.01.006

[15] Liu L, Yang M, Kang R, Wang Z, Zhao Y, et al (2011) HMGB1-induced autophagy promotes chemotherapy resistance in leukemia cells. Leukemia 25: 23–31. 20927132 10.1038/leu.2010.225

[16] White E, DiPaola RS (2009) The double-edged sword of autophagy modulation in cancer. Clin Cancer Res 15: 5308–5316. 19706824 10.1158/1078-0432.CCR-07-5023 PMC2737083

[17] Boya P, Gonzalez-Polo RA, Casares N, Perfettini JL, Dessen P, et al (2005) Inhibition of macroautophagy triggers apoptosis. Mol Cell Biol 25: 1025–1040. 15657430 10.1128/MCB.25.3.1025-1040.2005 PMC543994

[18] Goldberg SB, Supko JG, Neal JW, Muzikansky A, Digumarthy S, et al (2012) A phase I study of erlotinib and hydroxychloroquine in advanced non-small-cell lung cancer. J Thorac Oncol 7: 1602–1608. 22878749 10.1097/JTO.0b013e318262de4a PMC3791327

[19] Michaelides M, Stover NB, Francis PJ, Weleber RG (2011) Retinal toxicity associated with hydroxychloroquine and chloroquine: risk factors, screening, and progression despite cessation of therapy. Arch Ophthalmol 129: 30–39. 21220626 10.1001/archophthalmol.2010.321

[20] Khader M, Bresgen N, Eckl PM (2009) In vitro toxicological properties of thymoquinone. Food Chem Toxicol 47: 129–133. 19010375 10.1016/j.fct.2008.10.019

[21] Gali-Muhtasib H, Roessner A, Schneider-Stock R (2006) Thymoquinone: a promising anti-cancer drug from natural sources. Int J Biochem Cell Biol 38: 1249–1253. 16314136 10.1016/j.biocel.2005.10.009

[22] El-Mahdy MA, Zhu Q, Wang QE, Wani G, Wani AA (2005) Thymoquinone induces apoptosis through activation of caspase-8 and mitochondrial events in p53-null myeloblastic leukemia HL-60 cells. Int J Cancer 117: 409–417. 15906362 10.1002/ijc.21205

[23] Arafa el SA, Zhu Q, Shah ZI, Wani G, Barakat BM, et al (2011) Thymoquinone up-regulates PTEN expression and induces apoptosis in doxorubicin-resistant human breast cancer cells. Mutat Res 706: 28–35. 21040738 10.1016/j.mrfmmm.2010.10.007 PMC3037029

[24] Gurung RL, Lim SN, Khaw AK, Soon JF, Shenoy K, et al (2010) Thymoquinone induces telomere shortening, DNA damage and apoptosis in human glioblastoma cells. PLoS One 5: e12124. 20711342 10.1371/journal.pone.0012124 PMC2920825

[25] Shoieb AM, Elgayyar M, Dudrick PS, Bell JL, Tithof PK (2003) In vitro inhibition of growth and induction of apoptosis in cancer cell lines by thymoquinone. Int J Oncol 22: 107–113. 12469192

[26] Hosseinzadeh H, Parvardeh S (2004) Anticonvulsant effects of thymoquinone, the major constituent of Nigella sativa seeds, in mice. Phytomedicine 11: 56–64. 14971722 10.1078/0944-7113-00376

[27] Al-Majed AA, Al-Omar FA, Nagi MN (2006) Neuroprotective effects of thymoquinone against transient forebrain ischemia in the rat hippocampus. Eur J Pharmacol 543: 40–47. 16828080 10.1016/j.ejphar.2006.05.046

[28] Abe T, Wakimoto H, Bookstein R, Maneval DC, Chiocca EA, et al (2002) Intra-arterial delivery of p53-containing adenoviral vector into experimental brain tumors. Cancer Gene Ther 9: 228–235. 11896438 10.1038/sj.cgt.7700437

[29] Kondo E, Tanaka T, Miyake T, Ichikawa T, Hirai M, et al (2008) Potent synergy of dual antitumor peptides for growth suppression of human glioblastoma cell lines. Mol Cancer Ther 7: 1461–1471. 18566217 10.1158/1535-7163.MCT-07-2010

[30] Chazotte B (2008) Labeling lysosomes in live cells with fluorescent dyes for imaging. CSH Protoc 2008: pdb prot4929. 21356782 10.1101/pdb.prot4929

[31] Fischer AH, Jacobson KA, Rose J, Zeller R (2008) Mounting live cells attached to coverslips for microscopy. CSH Protoc 2008: pdb prot4927. 21356764 10.1101/pdb.prot4927

[32] Gali-Muhtasib H, Diab-Assaf M, Boltze C, Al-Hmaira J, Hartig R, et al (2004) Thymoquinone extracted from black seed triggers apoptotic cell death in human colorectal cancer cells via a p53-dependent mechanism. Int J Oncol 25: 857–866. 15375533

[33] Yang S, Wang X, Contino G, Liesa M, Sahin E, et al (2011) Pancreatic cancers require autophagy for tumor growth. Genes Dev 25: 717–729. 21406549 10.1101/gad.2016111 PMC3070934

[34] Degenhardt K, Mathew R, Beaudoin B, Bray K, Anderson D, et al (2006) Autophagy promotes tumor cell survival and restricts necrosis, inflammation, and tumorigenesis. Cancer Cell 10: 51–64. 16843265 10.1016/j.ccr.2006.06.001 PMC2857533

[35] Klionsky DJ, Abeliovich H, Agostinis P, Agrawal DK, Aliev G, et al (2008) Guidelines for the use and interpretation of assays for monitoring autophagy in higher eukaryotes. Autophagy 4: 151–175. 18188003 10.4161/auto.5338 PMC2654259

[36] Mizushima N, Yoshimori T (2007) How to interpret LC3 immunoblotting. Autophagy 3: 542–545. 17611390 10.4161/auto.4600

[37] Mizushima N, Yoshimori T, Levine B (2010) Methods in mammalian autophagy research. Cell 140: 313–326. 20144757 10.1016/j.cell.2010.01.028 PMC2852113

[38] Kroemer G, Jaattela M (2005) Lysosomes and autophagy in cell death control. Nat Rev Cancer 5: 886–897. 16239905 10.1038/nrc1738

[39] Gonzalez-Polo RA, Boya P, Pauleau AL, Jalil A, Larochette N, et al (2005) The apoptosis/autophagy paradox: autophagic vacuolization before apoptotic death. J Cell Sci 118: 3091–3102. 15985464 10.1242/jcs.02447

[40] Tiwari M, Bajpai VK, Sahasrabuddhe AA, Kumar A, Sinha RA, et al (2008) Inhibition of N-(4-hydroxyphenyl)retinamide-induced autophagy at a lower dose enhances cell death in malignant glioma cells. Carcinogenesis 29: 600–609. 18174255 10.1093/carcin/bgm264

[41] Shingu T, Fujiwara K, Bogler O, Akiyama Y, Moritake K, et al (2009) Inhibition of autophagy at a late stage enhances imatinib-induced cytotoxicity in human malignant glioma cells. Int J Cancer 124: 1060–1071. 19048625 10.1002/ijc.24030

[42] Ohkuma S, Poole B (1981) Cytoplasmic vacuolation of mouse peritoneal macrophages and the uptake into lysosomes of weakly basic substances. J Cell Biol 90: 656–664. 7287819 10.1083/jcb.90.3.656 PMC2111913

[43] Aki T, Nara A, Uemura K (2012) Cytoplasmic vacuolization during exposure to drugs and other substances. Cell Biol Toxicol 28: 125–131. 22431173 10.1007/s10565-012-9212-3

[44] Boya P, Kroemer G (2008) Lysosomal membrane permeabilization in cell death. Oncogene 27: 6434–6451. 18955971 10.1038/onc.2008.310

[45] Turk B, Turk V (2009) Lysosomes as “suicide bags” in cell death: myth or reality? J Biol Chem 284: 21783–21787. 19473965 10.1074/jbc.R109.023820 PMC2755904

[46] Kanzawa T, Bedwell J, Kondo Y, Kondo S, Germano IM (2003) Inhibition of DNA repair for sensitizing resistant glioma cells to temozolomide. J Neurosurg 99: 1047–1052. 14705733 10.3171/jns.2003.99.6.1047

[47] Cho CW, Choi DS, Cardone MH, Kim CW, Sinskey AJ, et al (2006) Glioblastoma cell death induced by asiatic acid. Cell Biol Toxicol 22: 393–408. 16897440 10.1007/s10565-006-0104-2

[48] Temme A, Herzig E, Weigle B, Morgenroth A, Schmitz M, et al (2005) Inhibition of malignant glioma cell growth by a survivin mutant retrovirus. Hum Gene Ther 16: 209–222. 15761261 10.1089/hum.2005.16.209

[49] Di Piazza M, Mader C, Geletneky K, Herrero YCM, Weber E, et al (2007) Cytosolic activation of cathepsins mediates parvovirus H-1-induced killing of cisplatin and TRAIL-resistant glioma cells. J Virol 81: 4186–4198. 17287256 10.1128/JVI.02601-06 PMC1866092

[50] Park CM, Park MJ, Kwak HJ, Lee HC, Kim MS, et al (2006) Ionizing radiation enhances matrix metalloproteinase-2 secretion and invasion of glioma cells through Src/epidermal growth factor receptor-mediated p38/Akt and phosphatidylinositol 3-kinase/Akt signaling pathways. Cancer Res 66: 8511–8519. 16951163 10.1158/0008-5472.CAN-05-4340

[51] Arafa el SA, Zhu Q, Barakat BM, Wani G, Zhao Q, et al (2009) Tangeretin sensitizes cisplatin-resistant human ovarian cancer cells through downregulation of phosphoinositide 3-kinase/Akt signaling pathway. Cancer Res 69: 8910–8917. 19903849 10.1158/0008-5472.CAN-09-1543 PMC3319094

[52] Chakravarti A, Erkkinen MG, Nestler U, Stupp R, Mehta M, et al (2006) Temozolomide-mediated radiation enhancement in glioblastoma: a report on underlying mechanisms. Clin Cancer Res 12: 4738–4746. 16899625 10.1158/1078-0432.CCR-06-0596

[53] El-Najjar N, Chatila M, Moukadem H, Vuorela H, Ocker M, et al (2010) Reactive oxygen species mediate thymoquinone-induced apoptosis and activate ERK and JNK signaling. Apoptosis 15: 183–195. 19882352 10.1007/s10495-009-0421-z

[54] Alhosin M, Ibrahim A, Boukhari A, Sharif T, Gies JP, et al (2012) Anti-neoplastic agent thymoquinone induces degradation of alpha and beta tubulin proteins in human cancer cells without affecting their level in normal human fibroblasts. Invest New Drugs 30: 1813–1819. 21881916 10.1007/s10637-011-9734-1

[55] Fan C, Wang W, Zhao B, Zhang S, Miao J (2006) Chloroquine inhibits cell growth and induces cell death in A549 lung cancer cells. Bioorg Med Chem 14: 3218–3222. 16413786 10.1016/j.bmc.2005.12.035

[56] Groth-Pedersen L, Ostenfeld MS, Hoyer-Hansen M, Nylandsted J, Jaattela M (2007) Vincristine induces dramatic lysosomal changes and sensitizes cancer cells to lysosome-destabilizing siramesine. Cancer Res 67: 2217–2225. 17332352 10.1158/0008-5472.CAN-06-3520

[57] Broker LE, Huisman C, Span SW, Rodriguez JA, Kruyt FA, et al (2004) Cathepsin B mediates caspase-independent cell death induced by microtubule stabilizing agents in non-small cell lung cancer cells. Cancer Res 64: 27–30. 14729603 10.1158/0008-5472.can-03-3060

[58] Mohanam S, Jasti SL, Kondraganti SR, Chandrasekar N, Lakka SS, et al (2001) Down-regulation of cathepsin B expression impairs the invasive and tumorigenic potential of human glioblastoma cells. Oncogene 20: 3665–3673. 11439329 10.1038/sj.onc.1204480

[59] Matteoni R, Kreis TE (1987) Translocation and clustering of endosomes and lysosomes depends on microtubules. J Cell Biol 105: 1253–1265. 3308906 10.1083/jcb.105.3.1253 PMC2114818

[60] Broker LE, Kruyt FA, Giaccone G (2005) Cell death independent of caspases: a review. Clin Cancer Res 11: 3155–3162. 15867207 10.1158/1078-0432.CCR-04-2223

[61] Koka PS, Mondal D, Schultz M, Abdel-Mageed AB, Agrawal KC (2010) Studies on molecular mechanisms of growth inhibitory effects of thymoquinone against prostate cancer cells: role of reactive oxygen species. Exp Biol Med (Maywood) 235: 751–760. 20511679 10.1258/ebm.2010.009369

[62] El-Najjar N, Ketola RA, Nissila T, Mauriala T, Antopolsky M, et al (2011) Impact of protein binding on the analytical detectability and anticancer activity of thymoquinone. J Chem Biol 4: 97–107. 22229047 10.1007/s12154-010-0052-4 PMC3124627

[63] Wang X, Thomas B, Sachdeva R, Arterburn L, Frye L, et al (2006) Mechanism of arylating quinone toxicity involving Michael adduct formation and induction of endoplasmic reticulum stress. Proc Natl Acad Sci U S A 103: 3604–3609. 16505371 10.1073/pnas.0510962103 PMC1450130

[64] Alinari L, Mahoney E, Patton J, Zhang X, Huynh L, et al (2011) FTY720 increases CD74 expression and sensitizes mantle cell lymphoma cells to milatuzumab-mediated cell death. Blood 118: 6893–6903. 22042694 10.1182/blood-2011-06-363879 PMC3568700

